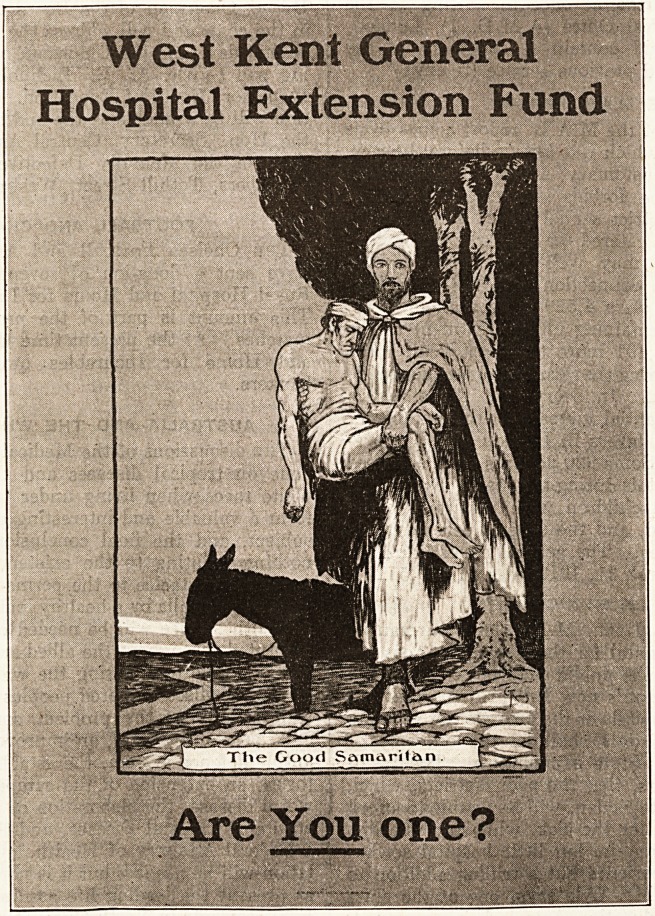# Hospital and Institutional News

**Published:** 1920-09-11

**Authors:** 


### HOSPITAL AND INSTITUTIONAL NEWS.

### NOTEWORTHY MEETINGS.

We understand that the Hon. Sir Arthur Stanley and Sir Napier Burnett will shortly travel north, visiting Birmingham and Manchester on September 16 and 17 respectively, when important meetings will be held, partly to inaugurate the new regional centres and partly to deal with the many questions arising out of Dr. Addison's new Act. We trust that valuable conclusions will be arrived at and discussions of the greatest general importance heard. In these districts, also, those directly or indirectly interested in this phase of medical and institutional administration should not fail to attend if circumstances will possibly permit.

### SHELL-SHOCK.

The abnormal number of neurasthenic cases caused during the war has led to the appointment by the War Office of a Committee to investigate the whole subject. It is understood that the inquiry is to be made on a purely scientific basis, and all available evidence will be examined by recognised neurological experts. Probably the Committee will begin its sittings almost immediately and they will be held in private. Officially they will "consider the different types of hysteria and traumatic neurosis, commonly called "shell-shock," collate the expert knowledge derived by the Service medical authorities and the medical profession from the experience of war with a view of recording for future use the ascertained facts as to its origin, nature, remedial treatment, and to advise whether by military training or education some scientific method of guarding against its occurrence cannot be devised."

### THE WEST KENT HOSPITAL POSTER.

A good poster must be seen to be believed. We therefore illustrate below one designed by Mr. C. Thomas, for the use of the West Kent General Hospital, Maidstone, which is conducting a county campaign in aid of its extension fund. The poster, entitled "The Good Samaritan," will be distributed throughout the villages. It shows a tall grave figure in Arab costume standing in front of a tree, with his dark donkey behind him, and in his arms the figure of an injured man. Printed on coffee-coloured paper with the figures outlined in black and relieved by indications of white as the turban and drapery, the figures stand against a blank evening sky and hold the eye with interest. Within the rim of the picture and at its foot is a panel inscribed "The Good Samaritan"; the black letters on the white ground form part of the composition. Above the picture on the brown paper border is the name of the hospital, and below it the question "Are you one?" carries the eye from the central figure to the name beneath. The whole is decorative and effective. It shows that a poster need not shout, but can hold the eye because of its intrinsic interest. We should like to see some more of Mr. Thomas's posters, and hope that this one will prove a useful ally to the hospital in the villages of Kent.

### MORE ARMY DOCTORS NEEDED.

The War Office has announced that it is prepared to grant temporary commissions in the Royal Army Medical Corps for a period of six months, for home service, to a limited number of medical men not over fifty-five years of age. Remuneration will be at the rate of £600 per annum for those who have not previously held commissions, and £650 for those who have served during the late war for a period of twelve months. In addition, they will receive free rations or an allowance in lieu when rations are not issued in kind, and the regulated allowances and expenses when travelling on duty. An outfit allowance of £30 will be made to those to whom it has not been previously issued. Applications should be made at once in writing to the Secretary of the War Office (A.M.D. 1), London, S.W. 1, and should contain a statement of the candidate's age and previous service (if any).

### THE M.A.B. REPORT.

The new issue of the M.A.B. report comes to us in the fuller form which was known before the war, and as was then customary, a fund of information is provided. Space forbids any extended review, but in that the district served is 121 square miles and the population catered for is nearly four and a-half millions, we may well note some of the major figures. In connection with the fever hospitals, admissions were 4,529 more than in 1918, and the number remaining under treatment at the end of the year 2,491 more than last year. The total admissions during the year ended December 31, 1919, were 21,962. In every way the statistics show a very substantial increase in the number of cases of infectious fevers in London over the previous three years. Some 920 patients were admitted to the mental hospitals during the year. During the same period 2,008 children were admitted to the homes of the board, and the tuberculosis hospitals received 3,206 cases. The general expenditure for the year ended March 31, 1919, was £1,469,512.

### STATE OR MUNICIPALITY?

Mr. C. E. Leo Lyle, M.P., the Chairman of Queen Mary's Hospital for the East End, does his best to secure that the public shall not overlook the fact that Dr. Addison's new Bill provides that the help to existing hospitals or the provision of separate rate-supported hospitals shall be the outcome of municipal and not State expenditure. The result will be, he considers, that the poor districts, which are already over-rate-ridden, will be unable to afford adequate provision for the sick, while the rich districts, which will require but little hospital accommodation, will experience but a trifling addition to the rates. Thus, Mr. Lyle says, one of the chief mistakes of the Education Acts is being repeated. He does not like either State or rate aid, but if we have to resort to one or the other, let the grants come from the State and not from the municipalities. He asks, What about the special hospitals? A district could hardly be expected substantially to maintain a hospital which received patients from all over the country. But would not a voluntary hospital make its claim upon the municipality which is responsible for the patient, and not upon that of the district? There will surely be a difference in principle as beween a voluntary hospital partly rate-supported and a municipal hospital.

### CARE OF THE MENTALLY DEFECTIVE.

A joint Conference of the Central Association for the Care of the Mentally Defective and the National Special Schools Union will be held at the Church House, Westminster, on Thursday, Friday, and Saturday, November 25, 26, and 27, 1920. The Rt. Hon. H. A. L. Fisher, President of the Board of Education, has promised, subject to his Parliamentary engagements permitting, to address the opening session, when Mr. Leslie Scott, K.C., M.P., will be in the chair. Sir William Byrne, Chairman of the Board of Control, will be in the chair and will address the Conference on the second day, and the Chairman on Saturday morning will be Dr. A. E. Eichholz, Chief Medical Inspector, Board of Education. Detailed information will be available at the end of September from the Hon. Secretary, Central Association for the Care of the Mentally Defective, Queen Anne's Chambers, Tothill Street, Westminster, S.W. 1.

### FOOTBALL AND CHARITY.

The Chelsea Football and Athletic Co., Ltd., have sent a donation of seventy guineas to the Royal Hospital and Home for Incurables, Putney. This amount is part of the proceeds of practice matches. At the present time the Royal Hospital and Home for Incurables owes £10,000 to its bankers.

### AUSTRALIA AND THE WHITE RACES.

The discussions of the Medical Congress at Brisbane on tropical diseases and the outlook of the white races when living under tropical conditions form a valuable and interesting contribution to the subject, and the final conclusion is that there is nothing pointing to the existence of inherent insuperable obstacles to the permanent occupation of tropical Australia by a healthy indigenous white race. A health service will be needed comparable to that instituted throughout the allied armies, which under similar conditions during the war and the absence of semi-civilised coloured peoples in Northern Australia simplifies the problem considerably. The chief points urged are, an improvement of the quarantine defence system, the establishment of laboratories, an extension of the campaign against hookworm disease, the elaboration of the Australian Institute of Tropical Disease, and the appointment of a Federal Ministry of Health. Considerable legislation will be needed, but it is noteworthy that even at present the leading life assurance offices do not treat residents in North Queensland differently from those in other parts of Australia, and the death-rate is lower for most diseases, while the birth-rate is the same. Infant mortality is favourable, and, generally speaking, tropical disease is scarce.

### CAPETOWN CHAIR OF PATHOLOGY.

The Chair of Pathology at Capetown University, which has been vacant since the death of Dr. Bla[illegible] Martin, has now been filled. Dr. Martin's successor is Dr. George Bertram Bartlett, B.A. (Cantab), M.R.C.S., (L.R.C.P., Assistant-Director of the Pathological Institute, London Hospital, and

Lecturer on Pathological Histology in the London Hospital Medical School. Dr. Bartlett has seen much active service on military duty. Commencing in 1914, he was successively pathologist to General Hospitals at Alexandria and Cairo, and, afterwards, Officer-in-Charge of the Malaria Department Laboratory in London. During 1918 and 1919 Dr. Bartlett was in charge of No. 4 Mobile Bacteriological Laboratory in East Africa.

### THE MINISTRY OF HEALTH AND DERBY SANATORIUM.

The Ministry of Health has informed the representatives of the Local War Pensions Committee, the Comrades of the Great War, and the National Federation of Discharged Soldiers at the recent inquiry concerning Derby Borough Sanatorium that it has made certain recommendations to improve conditions there. While stating that certain complaints laid before Dr. Chapman, the Ministry's medical officer, were incapable of proof or not substantiated, the Ministry found room for improvement, and in particular has recommended a reorganisation of the staff and better arrangements for storing, preparing, and distributing the food. Some of these improvements will involve structural alteration.

### A SUCCESSFUL FETE AT ROCHESTER.

An elaborate fête has been held at Rochester on behalf of (the local) St. Bartholomew's Hospital. Great pains were taken to make it a success, and much hard work was done by Mr. Featherstone, Mr. E. Blyth, and their staff of helpers. The proceeds were £1,742, and these may be increased by a few cheques which were conditionally promised. It is hoped also that the expenses may be met by a private donor. The aim was to raise £2,000, and this hope will be very nearly realised when the accounts are presented.

### THE NEW SANATORIUM FOR CONSUMPTIVE SAILORS.

When completed the King George's Sanatorium for Sailors, one of the establishments of the Seamen's Hospital Society, will accommodate 240 patients, and will be used exclusively for consumption. It is within a mile of Liphook, on the Hampshire downs, and the estate consists of 100 acres. The patients will be passed on from the Dreadnought Seamen's Hospital at Greenwich, the hospital at Albert Docks, and from the Hospital for Tropical diseases at Endsleigh Gardens. Cases of tubercle will be admitted at all stages of the disease. It is hoped that the first pavilions will be ready in the autumn.

### STOCKTON INFIRMARY FINANCES.

The satisfactory finances of Stockton and Thornaby Hospital are largely due to the generosity of the workmen, who, on their own initiative, have doubled their contributions. This should help Mr. H. G. Sanderson, the superintendent, when the approaching appeal to employers and the general public comes to be made. For the projected extensions, which include better accommodation for the nurses, a sum of £15,000 is in hand, thanks to the generosity of two persons—Sir Robert Ropner, who gave £10,000 in 1918, and Mr. and Mrs. C. W. Littleboy, who gave the remainder from the estate of their late son, who was lost in the war. This is a situation full of hope, and the appeal can hardly fail to be successful.

### THE GRANT FOR PENSIONERS' TREATMENT.

The renovation of the Royal Cornwall Infirmary, Truro, is proceeding, but the cost already exceeds the original estimate of £10,000, and further increases may be expected. A clause in the contract, for instance, makes the Hospital Committee responsible for any increase in wages while the work is being finished. At the same time, the hospital is not very seriously in debt, for the total deficit, according to the latest figures available, is £419, being the excess of expenditure for the past three years. Fifteen beds are reserved for pensioners under the County War Pensions Committee, and a sum of six shillings a day is paid for the maintenance of each pensioner. An increased grant is being asked for, so as to include remuneration for the medical staff, under the arrangements made after discussion with the British Hospitals Association, the B.M.A., and the Government. The Archdeacon of Cornwall, Canon Raffles Flint, is the new President of the hospital.

### UNIFORMITY OF LIVERPOOL CHARITY ACCOUNTS.

At the request of the Liverpool Council of Voluntary Aid a committee of prominent Liverpool accountants, all with experience in the audit of charity accounts, have issued a report on the question of uniformity of method in preparing and presenting the income and expenditure accounts and balance sheets of charities supported by general voluntary contributions. The report pays tribute to the work already done in this matter by the Burdett publications, King Edward's Hospital Fund for London, and King George's Fund for Sailors. It is cognisant of the probability that pressure will more and more be brought to bear by the Ministry of Health and other bodies to secure uniformity. No doubt the British Red Cross Society will accept King Edward's Fund's requirements for use in respect of provincial hospitals to which grants were made. All registered charities for the blind have to conform to the regulations of the Ministry of Health, and no doubt the Board of Education can influence semi-charitable schools for the deaf and dumb and others. There appears to be a real need for all controlling authorities to get together and prescribe forms of accounts which can be used in common by hospitals, schools, and miscellaneous charities, arranged in such a way to allow for detail additions to suit differences in the character of the work undertaken. The report is, apart from constructive suggestions, which in some respects may not suit all tastes of accountancy, is a useful record of what has been done, and may be done, to secure uniformity. It may be obtained at the Council's office, 14 Castle Street, Liverpool, at a cost of sixpence.

### FROM EAST AND WEST.

We have received annual reports from Toowoomba, Queensland, and from the Presbyterian Hospital, New York City. The Australian hospital analyses its expenditure under what the report describes as "Burdett's Classification," and is one of the many instances of how the Uniform System has reached to far-distant places. The average number of beds occupied at Toowoomba Hospital in 1919 was 132; the cost of each is not worked out, but, on a rough calculation, was a little over £2 a week. The Presbyterian Hospital, of New York, completes its financial year on September 30. Amongst the excellent illustrations in the well-printed report there is one of the group of hospital buildings in Park Avenue, very pleasantly situated, with good garden space on at least two sides. The report contains a sheet of figures which is a model of statistical thoroughness. It shows to decimal points of a cent the cost of a patient each day, in a private room or in a ward, under all headings. Out-patient department, visiting nursing, and educational and scientific work are treated in the same way, and the general result is very instructive. We notice that twenty nurses who served in the European War received British decorations and six the Croix de Guerre from the French Government.

### ANOTHER HOSPITAL AT CLAPHAM.

There is a possibility of yet another hospital being opened at Clapham. Negotiations are being completed for the purchase of a house in Crescent Lane, Clapham, to be used as a hospital for men and women. A community of voluntary nurses who have for many years had their headquarters in Barkham Terrace, Lambeth, are to take charge of the new institution as soon as the necessary details connected with the purchase of the premises are completed. These nurses have devoted themselves to visiting the sick poor in Camberwell, Peckham, Lambeth, Bermondsey and Southwark, and the hospital at Clapham will be a development of their sphere in South London.

### LEICESTER BASE HOSPITAL EXONERATED.

Certain allegations against the Ministry of Pensions Hospital have been examined by the Leicester War Pensions Committee. Mr. A. Sherriff, the chairman, had obtained an official report, which he presented, to show that the charges against the mortality rate were unfounded. This kind of statement would hardly be worth notice had not an ex-soldier explained that its object was to improve conditions, and in particular to revive various committees which had ceased to be active on the assumption that their work was done. The method cannot be recommended. It is always simpler to state definite grievances, and not to spoil a case by reckless over-statement in this way. The Disablement Sub-Committee is satisfied with the conditions.

### WEST RIDING V.A.D. SOUVENIR.

The West Riding Territorial Branch of the St. John Ambulance Association has issued an illustrated memorial book as a souvenir of the work of the V.A.D. hospitals in the West Riding. Besides describing the varied branches of the work of the detachment, the volume contains numerous photographs of the outside and inside of the different hospitals. Former members of the Yorkshire detachment who want copies should apply to the Assistant County Director's Office at 9 St. Leonard's, York.

### BRISTOL GENERAL HOSPITAL AND AMALGAMATION.

An agreement, the conditions of which render its value difficult to judge, has been come to by the Bristol General Hospital on the amended scheme for hospital amalgamation in the city. At a special meeting of the committee Mr. G. A. Wills, the president, proposed the following resolution, which was seconded by the chairman of the committee, Mr. Herbert M. Bother: "That the committee, having heard the views of the president, resolves that, subject to the assets of the institution being retained, agrees to the principle of the amended scheme, and that a Joint Council be set up for a period of three years, to be nominated as suggested, with the necessary modifications and with powers therein indicated, it being understood that the character of this institution as a general hospital shall be maintained." Since this resolution was passed unanimously, it would seem that the committee thinks the safeguards sufficient. Time alone can show how far the principle of amalgamation will be carried.

### A SUCCESSFUL EFFORT AT YARMOUTH.

The Special Appeal Committee of Yarmouth Hospital has succeeded in raising a sum of £600 by a trail of pennies, which weighed a ton and a quarter and stretched, we understand, nearly three miles. A good deal of organisation was required, for the plan involved a procession, numerous depôts for giving change, and numerous motor vehicles to gather the coppers. The counting took a day and a half, and most of Yarmouth took part to make the success of the occasion.

### THIS WEEK'S DRUG MARKET.

Some slight improvement in business is noticeable; this, for the most part, is made up of small orders, which, however, in the aggregate make a considerable total. It is too early to form an opinion whether the improvement is likely to be maintained for long, but provided the coal strike is averted, it is probable that trade generally will improve. Eucalyptus oil is attracting attention, as it always does at this season of the year; prices are higher, and it seems probable that they will advance further. So far as synthetic drugs are concerned there is no increase in the demand, while prices still have a tendency downwards. In consequence of a fall in the value of quicksilver makers of calomel, corrosive sublimate, white precipitate, and other salts of mercury have reduced their quotations. Bergamot, lemon, and orange oils are rather cheaper. The demand for cod-liver oil is quiet. The price of cream of tartar is lower. A fair business has been done in ipecacuanha at lower rates.

## Figures and Tables

**Figure f1:**